# One year monitoring of calcium release from an experimental composite containing calcium phosphate particles

**DOI:** 10.2340/biid.v12.44586

**Published:** 2025-08-28

**Authors:** Handially Vilela, Mariana Nakamura, Roberto Braga

**Affiliations:** Department of Biomaterials and Oral Biology, School of Dentistry, University of São Paulo, São Paulo, Brazil

**Keywords:** Calcium orthophosphate, ion release, resin composite

## Abstract

**Introduction:**

Ca^2+^ release from specimens made of a composite containing dicalcium phosphate dihydrate particles (CaHPO_4_.2H_2_O, dicalcium phosphate dihydrate [DCPD]) was followed during 1 year.

**Methods:**

Specimens were individually immersed in deionized water (*n* = 3). Every 2 weeks, immersion medium was collected and specimens were transferred to new vials with fresh medium. Ca^2+^ release was quantified using induced coupled plasma-optical emission spectrometry (ICP-OES). Data were analyzed by ANOVA/Tukey test (alpha: 5%).

**Results:**

Ca^2+^ release was observed during the entire 12-month period. Cumulative release was 1635.1 ± 145.3 μg/cm^2^ (179.7 ± 16.0 ppm), corresponding to 23.8 ± 2.0% of the total Ca mass in the specimen.

**Conclusion:**

The tested composite was capable of sustained Ca^2+^ release in water for 1 year. In spite of the limitations of this screening test, the results suggest that composites containing Calcium phosphate (CaP) particles could offer a long-term Ca^2+^ supply to the adjacent dental tissues.

KEY MESSAGES

1. The tested composite was capable of sustained Ca^2+^ release in water for 1 year.
2. The results suggest that composites containing CaP particles may offer a long-term Ca^2+^ supply to the adjacent dental tissues.

## Introduction

Restorative resin-based materials capable of ion release were developed with the purpose of promoting mineral precipitation at the tooth-restoration interface and prevent demineralization. Considering the increasing number of options commercially available, clinicians must know their ion release behaviour in order to make informed choices. The quantification of ions in different immersion media (e.g. water, artificial saliva – AS, simulated body fluid – SBF) does not reproduce the dynamics of the oral environment regarding pH, temperature, but still is a valuable screening tool often included in *in vitro* studies. These studies usually report ion release over short periods [1–3]. However, understanding how these materials behave over longer periods is important to define their clinical use.

Calcium phosphate (CaP) has been investigated as Ca^2+^ sources in resin composites for the last three decades [4]. Among CaP phases, dicalcium phosphate dihydrate (DCPD) presents highest solubility and refractive index similar to barium glass, which makes DCPD particles an interesting option regarding the material’s translucency and depth of cure [1, 2].

The purpose of this study was to establish the 12-month Ca^2+^ release curve of a DCPD-containing composite in water. The hypothesis was that release would gradually decrease, until no Ca^2+^ would be detected in the immersion medium.

## Methods

**Figure 1:**
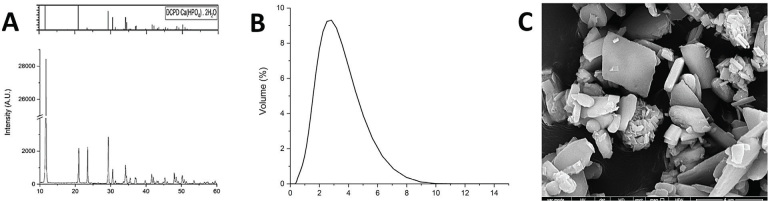
(A) Diffractogram confirming the formation of DCPD in the synthesis. (B) Particle size distribution (in µm). (C) DCPD particles observed under the scanning electron microscope (model 1010, JEOL, Tokyo, Japan).

DCPD particles were synthesized as described elsewhere [2]. Crystalline composition was confirmed by x-ray diffractometry (MultiFlex, Rigaku Corp., Tokyo, Japan). Particle median (D_50_) was 2.8 µm, as determined by laser light scattering (Mastersizer 2000, Malvern Instruments Ltd. Malvern, UK) (Figure 1).

A composite was formulated containing BisGMA and TEGDMA (1:1 in mols), with camphorquinone and EDMAB (ethyl-4-dimethylamino benzoate) added as photoinitiators (0.5wt% each). All chemicals were purchased from Sigma-Aldrich (St. Louis, MO, USA). DCPD particles (50wt%, 32.5vol%) were mechanically mixed to the resin (2,500 rpm for 1 min, Speedmixer DAC 150.1 FVZ-K, FlackTek Inc., Landrum, USA). The material was kept refrigerated until 2h prior to use.

Sample size calculation was performed using GPower 3.1.9.6 [5], using the parameters shown in Table 1. Specimens (5 × 1 mm, *n =* 3) were prepared with the use of a polyacetal mold. The material was photoactivated for 40 s (1,200 mW/cm^2^, Bluephase N, IvoclarVicadent, Schaan, Lieschtenstein). After 24 h dry storage at 37°C, specimen mass was determined in an analytical scale (model XS105, Mettler Toledo, Columbus, USA). The specimens were individually immersed in 5 mL of deionized water. After 24h and then every 2 weeks for 12 months, specimens were transferred to new tubes with fresh medium and the content of the previous tube was analyzed.

**Table 1:** Sample size calculation parameters.

Input parameters	Output parameters	
Effect size (partial η^2^): 0.940	Non-centrality parameter (λ): 45.925	Total sample size: 52
Significance level (α): 0.05	Critical F: 1.938	
Expected power (1-β): 0.80	Numerator df: 25	Actual power: 0.868
Number of groups: 26	Denominator df: 26	

Ca^2+^ release was quantified by inductively coupled plasma atomic emission spectrometry (ICP-OES, Agilent Technologies, Santa Clara, USA). Results were obtained in parts per million (ppm, or mg/L). The released Ca^2+^ mass per unit area of the specimen (μg/cm^2^) and the percentage release in relation to the Ca mass in the specimen were calculated. Data were homoscedastic (Brown-Forsythe test) and normally distributed (Shapiro-Wilk), and were analyzed using one-way ANOVA/Tukey test (α = 5%).

## Results

**Figure 2:**
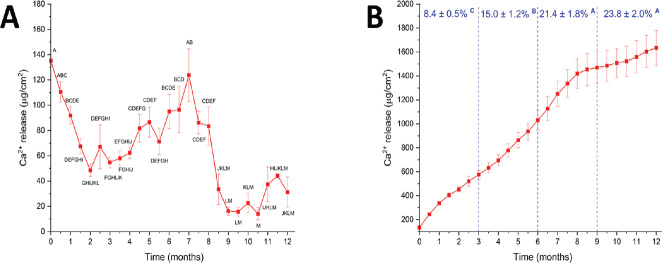
(A) Non-cumulative Ca^2+^ release in water (µg/cm^2^). (B) The same data shown in A are represented as cumulative values. Percentages represent the cumulative released Ca mass in relation to the Ca mass in the specimens in each trimester. Similar letters indicate a lack of statistically significant difference, one-way ANOVA/Tukey test (*p* > 0.05).

Non-cumulative Ca^2+^ release (Figure 2A) show a reduction in the first 2 months, followed by a gradual increase between 2 and 7 months. After 7 months, release decreased sharply (also indicated by the inflection in the cumulative curve in Figure 2B). In the last 3 months, average release was 30 ± 12 µg/cm^2^. At the end of 12 months, 23.8% of the Ca mass in the specimen was released.

## Discussion

A recent *in vitro* study verified that an experimental composite containing 30vol% DCPD (therefore, similar to the composite tested in this report) was able to promote significant mineral gain in dentin after 56 days [6]. Solvent uptake in resin-based materials occurs by two distinct mechanisms: gradient (Fickian) diffusion and relaxation-controlled swelling [7, 8]. Both mechanisms also characterize the transit of ions from the specimen to the surrounding medium [3]. Figure 2A suggests that at the initial stages Ca^2+^ release occurs by simple diffusion from DCPD particles at the specimen’s surface. Predictably, ion availability from these particles decreased with time. At the same time, polymer relaxation due to water sorption creates pathways for ionic diffusion from particles located in the specimen’s bulk. The shift from a mostly diffusion-controlled to a relaxation-controlled release mechanism after approximately 2 months agrees with findings of a previous study. Between 7 and 9 months, Ca^2+^ release decreased rapidly. Still, Ca^2+^ release was detected up to 12 months. Only 23.8% of the total Ca mass in the specimen was released. Considering that specimens were fully saturated, it means that the osmotic gradient is not strong enough to drive the ionic transit from the bulk of the specimen. Another possible explanation for the low efficiency of the ion-releasing process is the surface transformation of DCPD into less soluble phases.

As mentioned in the introduction, the quantification of ion release in solution does not reproduce the complex chemical interactions occurring at the tooth-material interface. The present investigation used water as immersion medium in order to maximize ion release, as other media (for instance, AF) contain ions that reduce the osmotic gradient. Phosphate release was not quantified as previous studies showed it is much lower than Ca due to its structural role in the DCPD crystal [9]. Considering that physiological fluids are supersaturated in respect to both ions, increasing the Ca^2+^ concentration will favour apatite precipitation [10]. Finally, it is noteworthy that the method showed high reproducibility in both sample preparation and spectrometric analysis, resulting in minimal data scatter and enabling the use of a small sample size, an important factor given the large number of experimental groups.

In spite of the limitations of this screening test, the present data contribute to elucidate the behavior of ion-releasing resin-based restorative materials over a long period of time, which is important to define their potential clinical uses. For instance, their higher release in the short-term (2–3 months) is interesting for its use as liner to accelerate the remineralization soft dentin in minimally invasive preparations [11], while long-term release may help postpone lesion development in high-risk patients [12] or around orthodontic brackets [13].

## Conclusion

In conclusion, the composite containing DCPD particles was capable of sustaining Ca^2+^ release for 1 year. The results suggest that after 2 months gradient diffusion was replaced by relaxation diffusion as the main mechanisms responsible for release. A sharp reduction in ion release occurred between 7 and 9 months. Ca^2+^ release can be considered a low-efficiency process, as only 23.8% of the Ca mass in the specimens was released after 1 year.

## Data Availability

Data set available on request.

## References

[1] Campos AL, Vela BF, Pires Silva Borges L, Trinca RB, Pfeifer CS, Braga RR. Compositional boundaries for functional dental composites containing calcium orthophosphate particles. J Mech Behav Biomed Mater. 2023;144:105928. 10.1016/j.jmbbm.2023.105928 37302206 PMC10330647

[2] Trinca RB, Oliveira BA, Vilela HDS, Braga RR. Effect of calcium orthophosphate particle size and CaP:glass ratio on optical, mechanical and physicochemical characteristics of experimental composites. Dent Mater. 2023;39(9):770–8. 10.1016/j.dental.2023.06.012 37423880

[3] Trinca RB, Vela BF, Dos Santos Vilela H, Braga RR. Ion release mechanisms in composites containing CaP particles and hydrophilic monomers. Dent Mater. 2024;40(7):1047–55. 10.1016/j.dental.2024.05.008 38772841

[4] Antonucci JM, Skrtic D, Eanes ED. Bioactive polymeric dental materials based on amorphous calcium phosphate. Polymer Preprints. 1994;35(2):460–1.

[5] Faul F, Erdfelder E, Lang AG, Buchner A. G*Power 3: a flexible statistical power analysis program for the social, behavioral, and biomedical sciences. Behav Res Methods. 2007;39(2):175–91. 10.3758/BF03193146 17695343

[6] Campos AL, Chiari M, Vela BF, Trinca RB, de Souza Balbinot G, Collares FM, et al. Dentin remineralization induced by experimental composites containing calcium orthophosphate particles. Dent Mater. 2025;41(3):265–71. 10.1016/j.dental.2024.12.004 39732611

[7] Sideridou ID, Achilias DS, Karabela MM. Sorption kinetics of ethanol/water solution by dimethacrylate-based dental resins and resin composites. J Biomed Mater Res B Appl Biomater. 2007;81(1):207–18. 10.1002/jbm.b.30655 16941599

[8] Berens AR, Hopfenberg HB. Diffusion and relaxation in glassy polymer powders .2. Separation of diffusion and relaxation parameters. Polymer. 1978;19(5):489–96. 10.1016/0032-3861(78)90269-0

[9] Rodrigues MC, Chiari MDS, Alania Y, Natale LC, Arana-Chavez VE, Meier MM, et al. Ion-releasing dental restorative composites containing functionalized brushite nanoparticles for improved mechanical strength. Dent Mater. 2018;34(5):746–55. 10.1016/j.dental.2018.01.026 29422326

[10] Bohner M, Lemaitre J. Can bioactivity be tested in vitro with SBF solution? Biomaterials. 2009;30(12):2175–9. 10.1016/j.biomaterials.2009.01.008 19176246

[11] Peters MC, Bresciani E, Barata TJ, Fagundes TC, Navarro RL, Navarro MF, et al. In vivo dentin remineralization by calcium-phosphate cement. J Dent Res. 2010;89(3):286–91. 10.1177/0022034509360155 20139340

[12] Melo MA, Weir MD, Rodrigues LK, Xu HH. Novel calcium phosphate nanocomposite with caries-inhibition in a human in situ model. Dent Mater. 2013;29(2):231–40. 10.1016/j.dental.2012.10.010 23140916 PMC3561736

[13] Uysal T, Amasyali M, Ozcan S, Koyuturk A, Akyol M, Sagdic D. In vivo effects of amorphous calcium phosphate-containing orthodontic composite on enamel demineralization around orthodontic brackets. Aust Dent J. 2010;55(3):285–91. 10.1111/j.1834-7819.2010.01236.x 20887516

